# Clinical Correlates, Ethnic Differences, and Prognostic Implications of Perivascular Spaces in Transient Ischemic Attack and Ischemic Stroke

**DOI:** 10.1161/STROKEAHA.117.016694

**Published:** 2017-05-22

**Authors:** Kui-Kai Lau, Linxin Li, Caroline E. Lovelock, Giovanna Zamboni, Tsz-Tai Chan, Man-Fung Chiang, Kin-Ting Lo, Wilhelm Küker, Henry Ka-Fung Mak, Peter M. Rothwell

**Affiliations:** From the Center for Prevention of Stroke and Dementia, Nuffield Department of Clinical Neurosciences, University of Oxford, United Kingdom (K.-K.L., L.L., C.E.L., G.Z., W.K., P.M.R.); Epsom and St Helier University Hospitals, NHS Trust, Carshalton, United Kingdom (C.E.L.); and Division of Neurology, Department of Medicine (K.-K.L., T.-T.C., M.-F.C., K.-T.L.) and Department of Diagnostic Radiology (H.K.-F.M.), Li Ka Shing Faculty of Medicine, University of Hong Kong.

**Keywords:** ischemic stroke, perivascular spaces, prospective studies, small vessel disease, transient ischemic attack

## Abstract

**Background and Purpose—:**

Perivascular spaces (PVSs) are considered markers of small vessel disease. However, their long-term prognostic implications in transient ischemic attack/ischemic stroke patients are unknown. Ethnic differences in PVS prevalence are also unknown.

**Methods—:**

Two independent prospective studies were conducted, 1 comprising predominantly whites with transient ischemic attack/ischemic stroke (OXVASC [Oxford Vascular] study) and 1 comprising predominantly Chinese with ischemic stroke (University of Hong Kong). Clinical and imaging correlates, prognostic implications for stroke and death, and ethnic differences in basal ganglia (BG) and centrum semiovale (CS) PVSs were studied with adjustment for age, sex, vascular risk factors, and scanner strength.

**Results—:**

Whites with transient ischemic attack/ischemic stroke (n=1028) had a higher prevalence of both BG and CS-PVSs compared with Chinese (n=974; >20 BG-PVSs: 22.4% versus 7.1%; >20 CS-PVSs: 45.8% versus 10.4%; *P*<0.0001). More than 20 BG or CS-PVSs were both associated with increasing age and white matter hyperintensity, although associations with BG-PVSs were stronger (all *P*<0.0001). During 6924 patient-years of follow-up, BG-PVSs were also independently associated with an increased risk of recurrent ischemic stroke (adjusted hazard ratio compared with <11 PVSs, 11–20 PVSs: HR, 1.15; 95% confidence interval, 0.78–1.68; >20 PVSs: HR, 1.82; 1.18–2.80; *P*=0.011) but not intracerebral hemorrhage (*P*=0.10) or all-cause mortality (*P*=0.16). CS-PVSs were not associated with recurrent stroke (*P*=0.57) or mortality (*P*=0.072). Prognostic associations were similar in both cohorts.

**Conclusions—:**

Over and above ethnic differences in frequency of PVSs in transient ischemic attack/ischemic stroke patients, BG and CS-PVSs had similar risk factors, but although >20 BG-PVSs were associated with an increased risk of recurrent ischemic stroke, CS-PVSs were not.

## Abstract 2

Supplemental Digital Content is available in the text.

Perivascular spaces (PVSs) are tiny cavities of cerebrospinal fluid that surround arterioles that penetrate the brain parenchyma.^1^ They are most frequently found in the inferior basal ganglia (BG), centrum semiovale (CS), and midbrain.^2^ Although it is normal to have a few visible PVSs on neuroimaging,^3^ an increased burden of BG and CS-PVSs has been associated with increasing age,^4–7^ hypertension,^4–6,8^ renal impairment,^9^ white matter hyperintensity (WMH),^4–6,8,10^ and lacunes.^4,6,8,10^ BG-PVSs in addition have also been associated with male sex,^6^ mean systolic and diastolic blood pressure,^8,11^ deep or infratentorial cerebral microbleeds,^7,8^ and also stroke caused by small vessel occlusion.^10^ BG-PVSs have been considered a marker of hypertensive arteriopathy secondary to endothelial dysfunction^7,8^ and have recently been proposed as 1 of the 4 components of the Total Small Vessel Disease (SVD) Score.^12^ In contrast, CS-PVSs have been associated with lobar microbleeds in healthy adults and in those with cognitive impairment.^5,8^ A high burden of CS-PVSs has also been noted in patients with cerebral amyloid angiopathy (CAA).^7,13^ It has, therefore, been hypothesized that in contrast to BG-PVSs, CS-PVSs may be a neuroimaging marker of CAA by representing fluid and metabolic waste clearance dysfunction because of vascular amyloid deposition.^7,8,14^

Although PVSs have shown potential as an imaging biomarker of hypertensive angiopathy and CAA, the long-term prognostic implications of PVSs among patients with transient ischemic attack (TIA) and ischemic stroke have yet to be determined. Ethnic differences in PVS are also uncertain. To address these unanswered questions, we performed 2 large prospective studies, consisting of >2000 whites and Chinese with TIA/ischemic stroke from 2 independent cohorts to determine the associations of PVSs with ethnicity, vascular risk factors, other neuroimaging markers of SVD, and long-term risks of stroke and death.

## Methods

We prospectively studied patients with TIA/ischemic stroke from 2 cohorts: the OXVASC (Oxford Vascular) Study and the University of Hong Kong (HKU). In brief, OXVASC is an ongoing population-based study of all acute vascular events occurring within a population of all 92 728 individuals, irrespective of age, who are registered with 100 general practitioners in 9 general practices of Oxfordshire, United Kingdom.^15^ The analysis herein includes 1080 consecutive cases of TIA/ischemic stroke recruited from November 1, 2004, to September 30, 2014, who had a cerebral magnetic resonance imaging (MRI). The imaging protocol of OXVASC has been described in detail elsewhere.^16^ Briefly, from April 1, 2002, to March 31, 2010 (phase 1), MRI and magnetic resonance angiography were done in selected patients when clinically indicated. From April 1, 2010, onward (phase 2), brain MRI and magnetic resonance angiography of intra- and extracranial vessels became the first-line imaging methods.^16^ A further 1076 consecutive patients who were predominantly Chinese with a diagnosis of acute ischemic stroke who received an MRI scan and magnetic resonance angiography of the intra- and extracranial blood vessels at the HKU MRI Unit were recruited during March 1, 2008, to September 30, 2014.

We collected demographic data, atherosclerotic risk factors, and details of hospitalization of index event during face-to-face interview and cross-referenced these with primary care records and hospital records in both cohorts. Cause of TIA/ischemic stroke was classified according to the modified Trial of ORG 10172 in Acute Stroke Treatment (TOAST) criteria.^17^

Details of scan parameters are documented in Table I in the online-only Data Supplement. Two neuroradiologists (H.K.F.M. and W.K.) supervised the interpretation of the MRI images. PVSs were defined as small (<3 mm) punctate (if perpendicular to the plane of scan) or linear (if longitudinal to the plane of scan) hyperintensities on T2 images in the BG or CS based on a previously validated scale.^18^ In patients with asymmetrical number of PVSs, the side with the higher number of PVSs was counted.^18^ Burden of PVSs was then stratified into 3 groups: <11, 11 to 20, and >20 (frequent–severe).^18^ Definitions of subcortical and periventricular WMH, microbleeds, and lacunes are provided in the online-only Data Supplement. The intrarater *κ* for burden of PVS (<11, 11–20, and >20) was 0.86 (BG) and 0.84 (CS) in OXVASC and 0.86 (BG) and 0.72 (CS) in HKU (50 scans in each center). Seventy-five MRI scans from HKU were cross-interpreted by investigators in OXVASC with an interrater *κ* of 0.64 for both BG and CS-PVSs.

All patients in OXVASC were followed up regularly by a research nurse or physician after 1, 3, 6, 12, 24, 60, and 120 months after the index event. Patients recruited from HKU were followed up by a clinician every 3 to 6 months, or more frequently if clinically indicated. All patients were assessed for recurrent stroke (ischemic and hemorrhagic) and death (vascular and nonvascular; see definitions in the online-only Data Supplement). Where needed, details of clinical outcomes were supplemented by electronic or paper medical records from individual primary care practices, hospitals, and the Deaths General Register Office.

Patients gave written informed consent after an event or assent was obtained from relatives for patients who were unable to provide consent. Both cohorts were approved by the local research ethics committee.

### Statistical Analysis

We compared differences in baseline and imaging characteristics in the OXVASC and HKU cohorts using Student *t* test for continuous variables and χ^2^ test for categorical variables. The predictors of >20 BG and CS-PVSs were determined using a logistic regression model adjusted for center and MRI scanner strength. Variables including age, male sex, vascular risk factors (hypertension, hyperlipidaemia, diabetes mellitus, smoking, and atrial fibrillation), renal impairment (defined as glomerular filtration rate <60 mL/min/1.73 m^2^, as measured by the Modification of Diet in Renal Disease Study equation^19^), periventricular and subcortical WMH, deep and lobar microbleed number and lacunes were entered into a univariate analysis model. All variables were subsequently entered into a multivariate analysis model to determine the independent predictors of >20 BG and CS-PVSs. The multivariate model to determine the independent predictors of >20 BG-PVSs was also adjusted for CS-PVSs and vice versa.

In a logistic regression model, we determined the odds of a TIA/ischemic stroke because of SVD with increasing burden of BG and CS-PVSs, adjusted for age, sex, vascular risk factors, center, and MRI scanner strength.

We used Kaplan–Meier survival analysis to calculate the 5-year risk of a recurrent stroke (ischemic and hemorrhagic) and all-cause mortality, censored at death or March 31, 2015, according to the burden of PVSs. We also determined, by Cox regression analysis, the unadjusted and adjusted (for age, sex, vascular risk factors, center, and MRI scanner strength) risks of recurrent stroke (ischemic and hemorrhagic), mortality (vascular and nonvascular) in patients with 11 to 20 and >20 BG and CS-PVSs compared with <11 PVSs as reference. Finally, we performed a stratified analysis to determine whether the prognosis of PVSs differed in patients with no or mild versus moderate or severe periventricular and subcortical WMH.

All analyses were done with SPSS version 20.

### Role of the Funding Source

The funding source had no role in study design, data collection, data analysis, data interpretation, or writing of the report. The corresponding authors had full access to all the data in the study and had the final responsibility for the decision to submit for publication.

## Results

The 2 study populations contributed a total of 2156 patients. After excluding 154 patients with incomplete clinical or imaging data, 2002 (OXVASC n=1028, 542 TIA, 486 ischemic stroke; HKU n=974, all ischemic stroke) were included in the final analysis. Baseline clinical and imaging characteristics of patients are shown in Table 1. Proportion of patients according to TOAST classification is shown in Table II in the online-only Data Supplement. HKU patients had a higher proportion of men (*P*=0.001) and were more likely to have hypertension and diabetes mellitus (*P*<0.0001), whereas OXVASC patients were more likely to have hyperlipidaemia or a history of smoking (*P*<0.0001; Table 1).

Patients from OXVASC had a higher burden of >20 BG (22.4% versus 7.1%; *P*<0.0001) and CS-PVSs (45.8% versus 10.4%; *P*<0.0001) compared with those from HKU (Table 1). These differences in PVS burden remained, despite stratification of individuals into stroke subtypes (Table III in the online-only Data Supplement). OXVASC patients also had more severe periventricular WMH (*P*<0.0001; Table 1). In contrast, those from HKU had a greater burden of subcortical WMH (*P*<0.0001) and microbleeds (*P*<0.0001; Table 1). These differences remained in analyses confined to patients who received an MRI with a 3T scanner (Table IV in the online-only Data Supplement). However, within OXVASC, patients who received a 3T MRI (n=446) had a greater burden of >20 BG-PVSs (25.8% versus 19.8%; *P*=0.022) and CS-PVSs (55.6% versus 38.3%; *P*<0.0001) compared with patients who received a 1.5T MRI (n=582).

**Table 1.**
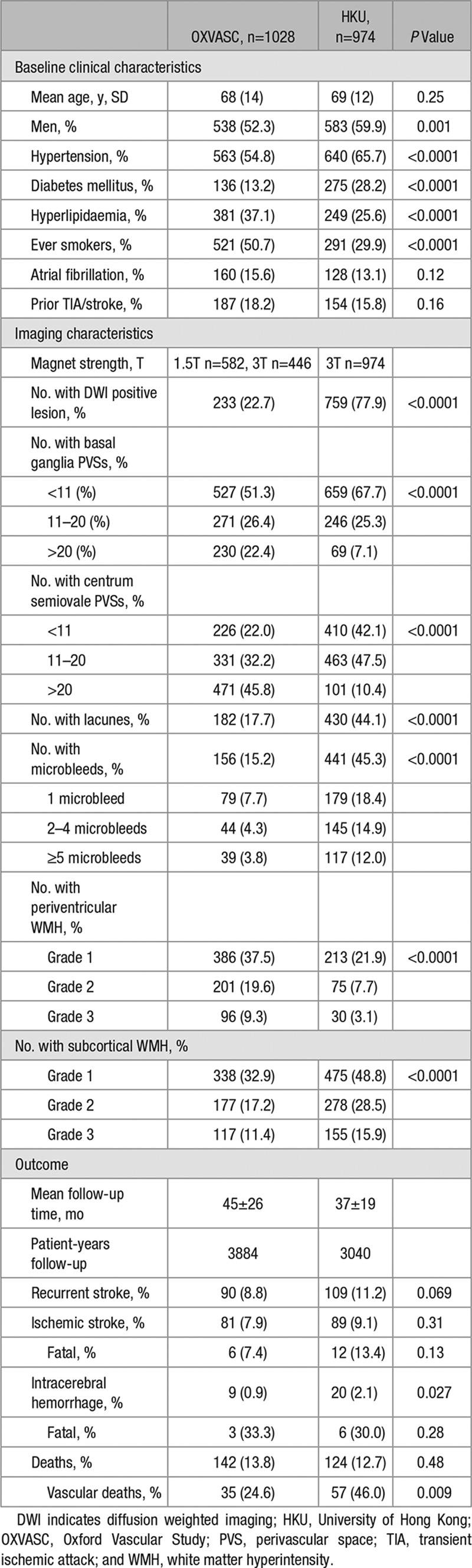
Clinical and Imaging Characteristics of the Study Population

Burden of BG and CS-PVSs increased with age, baseline history of hypertension, atrial fibrillation, and renal impairment (*P*<0.05; Table V in the online-only Data Supplement). Burden of BG and CS-PVSs was also greater in patients with lacunes and severe WMH (*P*<0.05; Table V in the online-only Data Supplement). In a multivariate analysis, >20 BG or CS-PVSs were associated with increasing age (multivariate adjusted odds ratio [OR], BG: 1.05; 95% confidence interval [CI], 1.03–1.07; *P*<0.0001; CS: OR, 1.01; 95% CI, 1.00–1.03; *P*=0.020) and subcortical WMH (BG: OR, 1.44; 95% CI, 1.20–1.72; *P*<0.0001; CS: OR, 1.28; 95% CI, 1.09–1.50; *P*=0.003). More than 20 BG-PVSs were also associated with atrial fibrillation (OR, 1.58; 95% CI, 1.10–2.29; *P*=0.014) and periventricular WMH (OR, 2.01; 95% CI, 1.66–2.44; *P*<0.0001; Table 2; Table VI in the online-only Data Supplement). Although underlying significant (>50%) large artery atherosclerosis was not related to >20 BG-PVSs (multivariate adjusted OR, 1.10; 95% CI, 0.77–1.56; *P*=0.61), an independent association between >20 CS-PVSs with significant large artery disease was noted (OR, 1.44; 95% CI 1.07–1.93; *P*=0.015). Whites, as compared with Chinese, were at increased odds of >20 BG (multivariate adjusted OR, 2.09; 95% CI, 1.35–3.22; *P*=0.001) and CS-PVSs (OR, 8.82; 95% CI, 6.25–12.46; *P*<0.0001). These ORs remained similar after additional adjustment of MRI magnet strength (BG: OR, 2.50; 95% CI, 1.56–4.02; *P*=0.0002; CS: OR, 11.93; 95% CI, 8.15–17.47; *P*<0.0001).

**Table 2.**
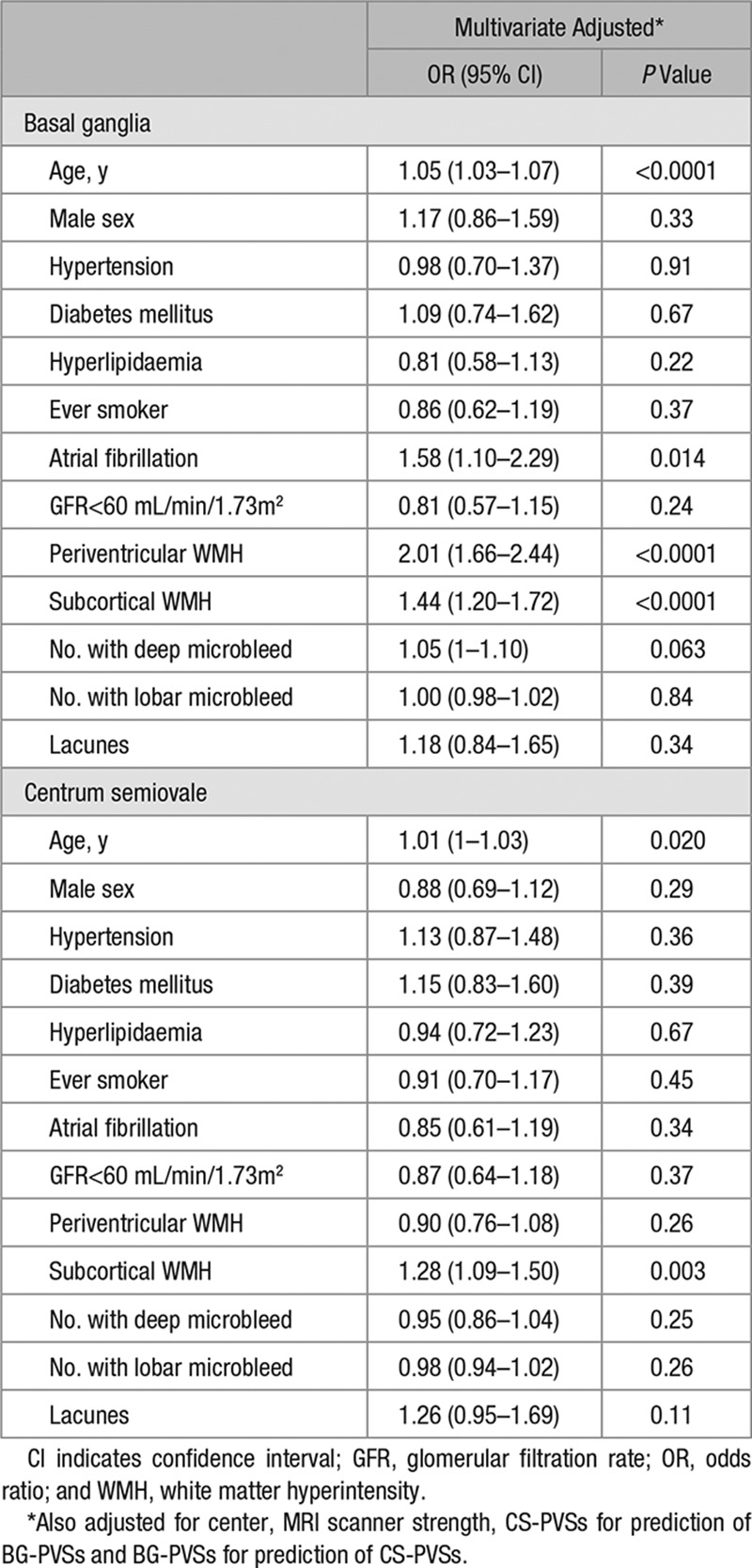
Clinical Correlates of >20 Perivascular Spaces

A 26.8% of the study population was classified to have TIA/ischemic stroke because of SVD (Table II in the online-only Data Supplement). These patients were associated with a higher BG and CS-PVS burden (multivariate adjusted OR compared with <11 PVSs, 11–20 BG-PVSs: OR, 2.44; 95% CI, 1.45–4.10; >20 BG-PVSs: OR, 2.82; 95% CI, 1.60–4.97; *P*=0.0002; 11–20 CS-PVSs: OR, 2.54; 95% CI, 1.32–4.88; >20 CS-PVSs: OR, 4.20; 95% CI, 2.19–8.06; *P*<0.0001).

**Figure 1.**
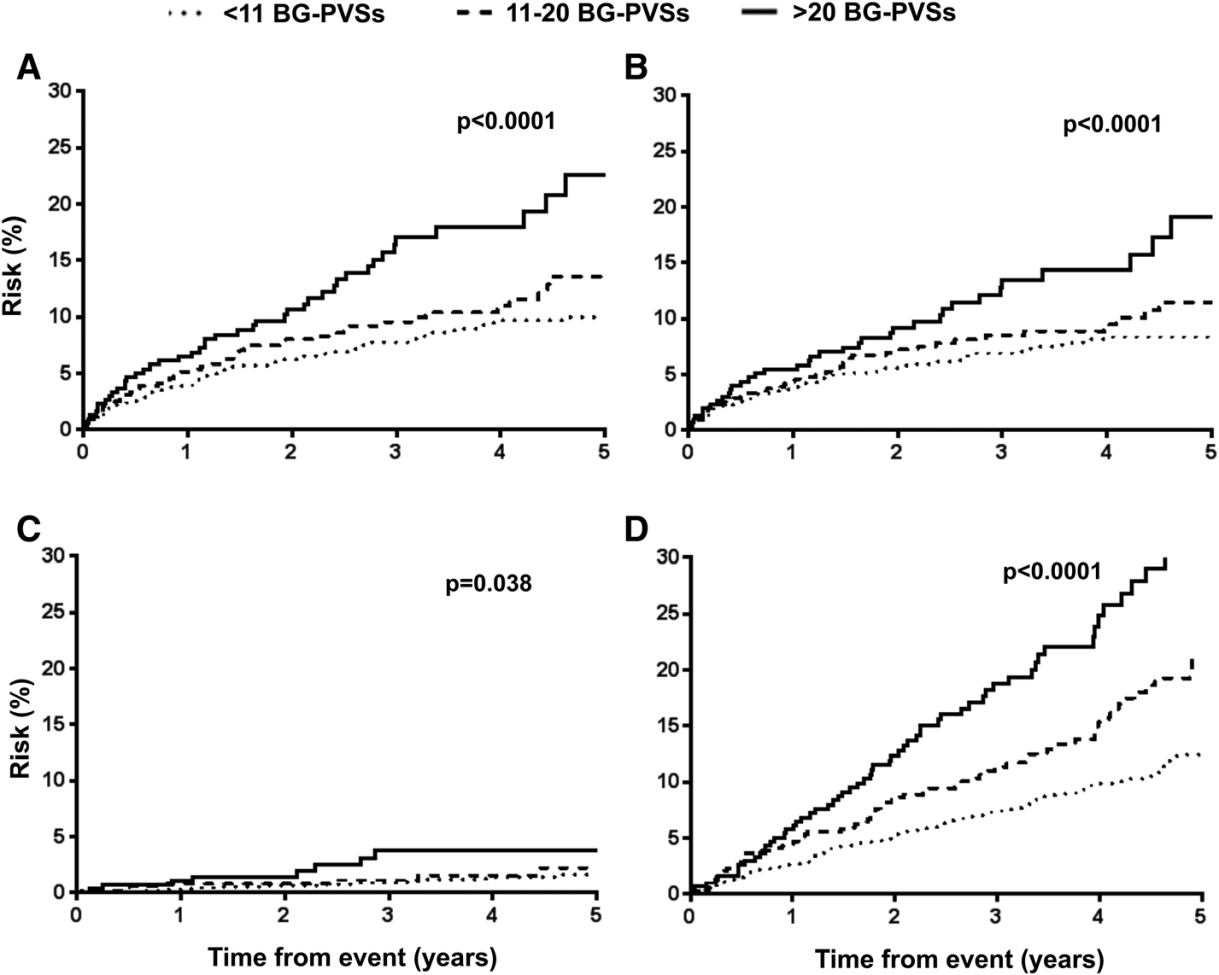
Risk of (**A**) recurrent stroke, (**B**) recurrent ischemic stroke, (**C**) intracerebral hemorrhage, and (**D**) all-cause mortality among transient ischemic attack/ischemic stroke patients with increasing basal ganglia perivascular space burden. BG-PVS indicates basal ganglia perivascular spaces.

**Figure 2.**
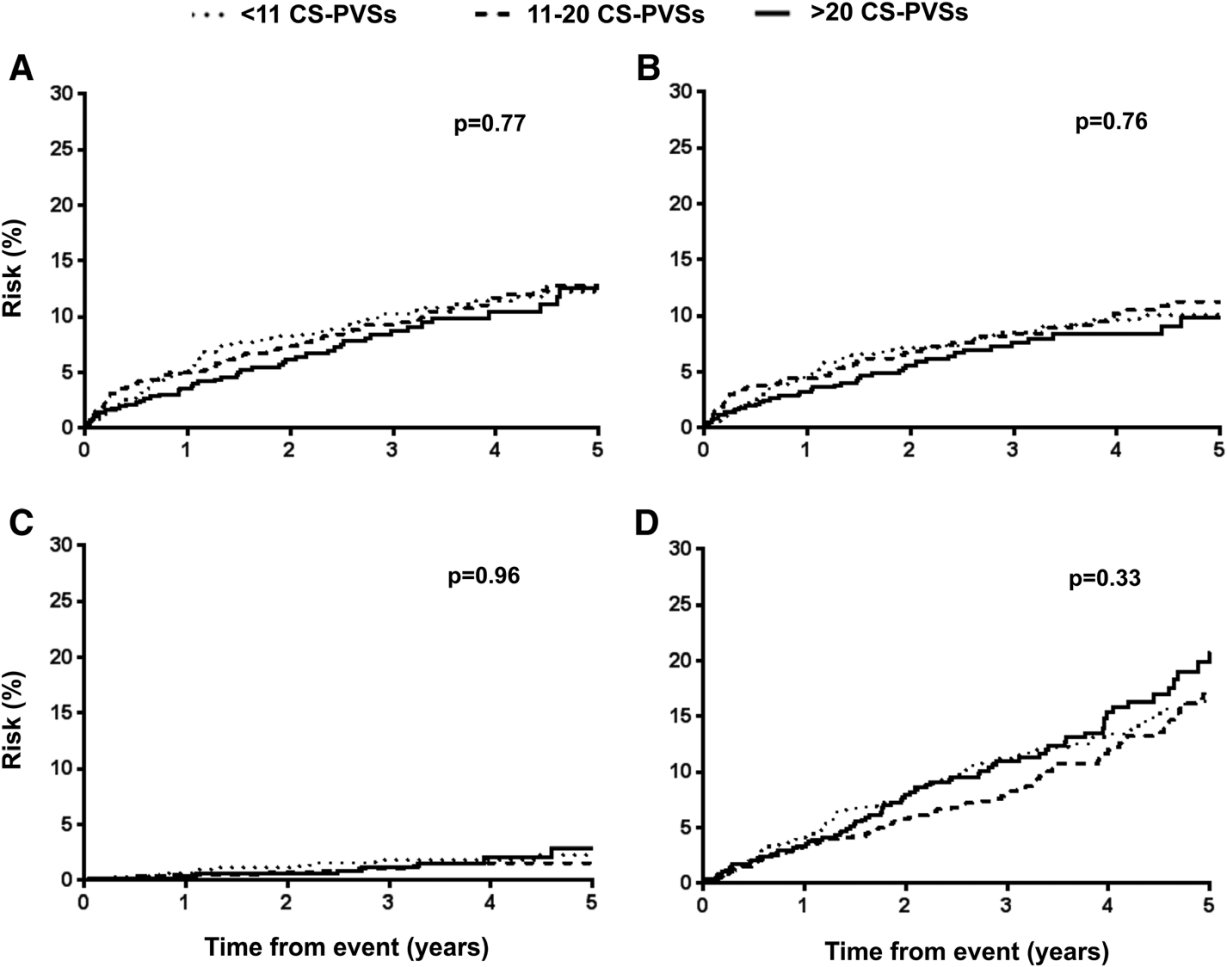
Risk of (**A**) recurrent stroke, (**B**) recurrent ischemic stroke, (**C**) intracerebral hemorrhage, and (**D**) all-cause mortality among transient ischemic attack/ischemic stroke patients with increasing centrum semiovale perivascular space burden. CS-PVS indicates centrum semiovale perivascular spaces.

After a mean follow-up of 42±23 months (OXVASC 45±26 months, HKU 37±19 months, 6924 patient-years of follow-up), 199 recurrent strokes occurred (85.4% ischemic; Table 1). Two hundred sixty-six patients died, 34.6% of which were vascular deaths. The 5-year risk of recurrent ischemic stroke and intracerebral hemorrhage (ICH) in patients with <11, 11 to 20, and >20 BG-PVSs was 8.5%, 11.5%, and 19.3% (log-rank test: *P*<0.0001) and 1.6%, 2.3%, and 3.7%, respectively (*P*=0.038; Figure 1). An increasing burden of BG-PVSs was also associated with a higher all-cause mortality (*P*<0.0001; Figure 1). In contrast, burden of CS-PVSs was not associated with recurrent ischemic stroke (*P*=0.76), intracerebral hemorrhage (ICH; *P*=0.96), or all-cause mortality (*P*=0.33; Figure 2).

On Cox regression analysis, strong univariate association between increasing burden of BG-PVSs with all-cause mortality was noted (*P*<0.0001), but this association disappeared after adjustment for age and sex (*P*=0.058) and on multivariate adjustment (*P*=0.16; Table VII in the online-only Data Supplement). In contrast, the strong univariate associations between increasing burden of BG-PVSs with recurrent ischemic stroke persisted with multivariate adjustment (hazard ratio compared with <11 BG-PVSs, 11–20: HR, 1.15; 95% CI, 0.78–1.68; >20: HR, 1.82; 95% CI, 1.18–2.80; *P*=0.011; Table 3; Table VIII in the online-only Data Supplement). BG-PVS burden was not independently associated with ICH (*P*=0.10; Table 3). An increasing burden of CS-PVSs was not related to ischemic stroke (*P*=0.42), ICH (*P*=0.69), or mortality (*P*=0.072; Table 3; Table VII in the online-only Data Supplement). When patients were stratified by MRI scanner in OXVASC, the prognostic value of BG and CS-PVSs remained similar for prediction of recurrent stroke (BG-PVS: *P*=0.15; CS-PVS: *P*=0.45; Table IX in the online-only Data Supplement). Similarly, no heterogeneity was observed when analyses for risk of recurrent stroke were stratified by patients with no or mild versus moderate–severe WMH (BG-PVS: *P*=0.92; CS-PVS: *P*=0.076; Table X in the online-only Data Supplement). In an unadjusted model, burden of BG-PVSs, microbleeds, periventricular WMH, subcortical WMH, and presence of lacunes were all associated with recurrent ischemic stroke (*P*<0.05; Table XI in the online-only Data Supplement). Forward stepwise multivariate Cox regression model adjusting for all neuroimaging markers of SVD revealed that burden of BG-PVSs (*P*=0.001) and microbleeds (*P*=0.001) as independent predictors of recurrent ischemic stroke (Table XI in the online-only Data Supplement).

**Table 3.**
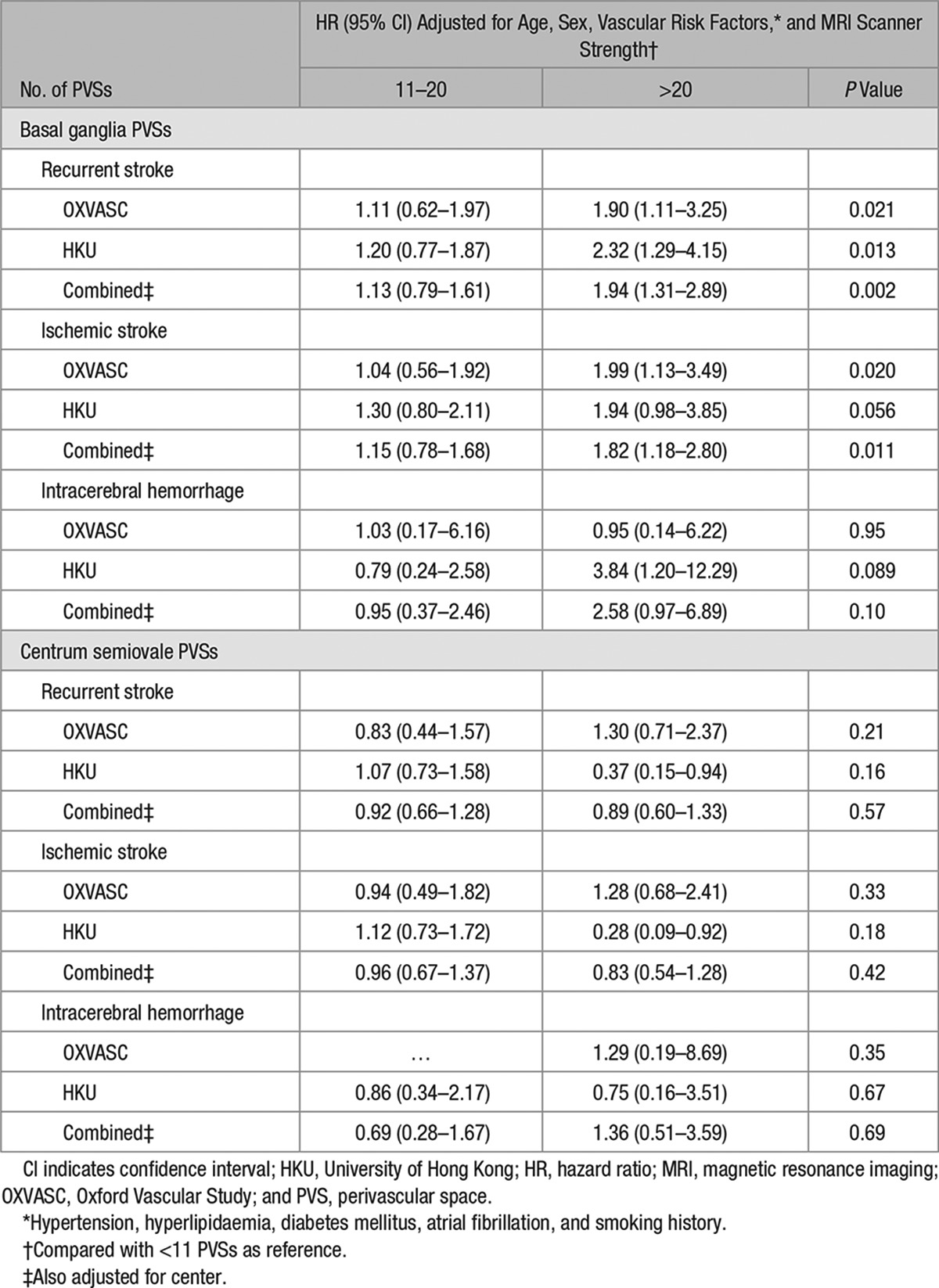
Cox Regression Analyses of Recurrent Stroke With Increasing Burden of Perivascular Spaces Versus <11 Perivascular Spaces

## Discussion

Our study has combined the 2 largest current cohorts from the west and the east of the clinical implications of BG and CS-PVSs in patients with TIA/ischemic stroke and is the first to determine the ethnic differences in prevalence. In our study, PVSs were coded according to a validated rating scale,^18^ with excellent intrarater variability and good interrater variability when scans were cross-interpreted between the 2 centers. Our study is also the first to determine the long-term prognostic implications of PVSs in patients with TIA/ischemic stroke.

Our results support those from previous studies that BG and CS-PVSs are both markers of hypertensive angiopathy.^6,8,14^ We too found that PVSs were associated with age^4–6^ and WMH.^5,6^ Concordant with previous studies,^4–8,10^ we also found that compared with CS-PVSs, BG-PVSs were a stronger marker of hypertensive angiopathy, with greater associations with periventricular and subcortical WMH and that BG-PVSs were more strongly associated with TIA/ischemic stroke because of SVD. Although previous studies have also noted significant associations of BG-PVS with deep microbleeds,^7,8^ this finding did not reach statistical significance in our cohorts (*P*=0.063).

The stronger association of BG-PVSs with hypertensive angiopathy was also reflected in our long-term follow-up data. Compared with <11 PVSs, TIA/ischemic stroke patients with >20 BG-PVSs were at 1.8-fold increased risk of recurrent ischemic stroke on multivariate analysis. There was a trend toward patients with increasing burden of BG-PVSs being similarly at increased risk of subsequent ICH and mortality. Furthermore, we were able to demonstrate that the prognostic implications of BG-PVSs were independent of other neuroimaging markers of SVD.

In contrast, although previous studies have revealed an association of CS-PVSs with lobar microbleeds^5,8^ and CAA,^7^ suggesting that CS-PVSs may be an imaging biomarker of CAA,^5,7,8^ CS-PVSs were not associated with lobar microbleeds nor adverse clinical events including ICH in our cohorts. It should be noted, however, that studies that have ascertained the relationship of CS-PVSs with lobar microbleeds were based on either healthy adults or subjects recruited from a memory clinic,^5,8^ with an expected lower prevalence of vascular risk factors and hence less severe imaging markers of SVD compared with patients in our study. It is widely accepted that PVSs may be difficult to identify in patients with extensive WMH.^18^ This is particularly the case for CS-PVSs that are often masked by subcortical WMHs. Indeed, in our high-risk cohort, where 36% of individuals had moderate–severe subcortical WMH, the true prevalence of CS-PVSs would without doubt be underestimated.

Our results also demonstrate that significant ethnic differences in PVS prevalence exist. We showed a similar prevalence of >20 BG and CS-PVSs in OXVASC to a previous study of whites with TIA/ischemic stroke.^4^ In contrast, however, our study showed that Chinese with TIA/ischemic stroke had a much lower prevalence of PVSs. The prevalence of BG and CS-PVSs among Chinese with ischemic stroke has previously been reported.^9,20^ One study showed that 10.7% of subjects had >40 BG-PVSs,^20^ and in another, ≈40% of subjects had >10 BG or CS-PVSs.^9^ These 2 studies, however, were purely based on patients with lacunar stroke subtype.^9,20^ In a large cohort of neurologically healthy Japanese individuals, a low prevalence of >20 BG-PVS and CS-PVS of 2.5% and 22.6% was similarly noted.^8^ Such low prevalence of >20 PVSs in the HKU cohort would have limited the statistical power when determining the clinical correlates of PVSs and attributed to some of the differences observed when compared with OXVASC. Atrial fibrillation was also noted to be significantly associated with >20 BG-PVSs, whereas underlying large artery disease was significantly associated with >20 CS-PVSs in our cohorts. Further studies to confirm and to delineate the underlying mechanisms of our findings would be required. Finally, our findings are also limited by patients in OXVASC being scanned on 4 different scanners during the 10-year study period. However, although this could have been a potential source of heterogeneity, the prognostic values for prediction of recurrent stroke with increasing burden of PVSs were similar across the 4 scanners, suggesting that the prognostic value of PVSs is robust to variations in scanner type and sequences. PVS size, symmetry, or ventricular size was not studied in our cohorts. Hence, we were only able to study clinical and imaging correlates and prognostic implications according to PVS number^18^ but not its size or symmetry.

Our study has several clinical implications. First, in 2 large cohorts, our results confirm BG-PVSs as a marker of SVD, independent of WMH. These results, therefore, justify the inclusion of BG-PVSs into the recently derived Total SVD Score.^12^ In the current version,^12^ patients with >11 BG-PVSs are given 1 point, as are patients with severe periventricular WMH or moderate–severe subcortical WMH. Whether alternative cutoffs (eg, >20 BG-PVSs) should be used instead in view of the relatively low prognostic value of patients with 11 to 20 BG-PVSs noted in our study would require further research. Second, although the burden of CS-PVSs may possibly have prognostic implications in healthy individuals or those seen in the Memory Clinic, the role of CS-PVSs as a prognostic imaging marker in the TIA/ischemic stroke population seems to be limited.

In conclusion, in addition to identifying ethnic differences in frequency of PVSs, we found that BG-PVSs are markers of hypertensive angiopathy and predict risk of recurrent ischemic stroke in patients with TIA/ischemic stroke, independent of WMH. In contrast, the prognostic value of CS-PVSs in TIA/ischemic stroke is limited.

## Acknowledgments

We acknowledge the use of the facilities of the Acute Vascular Imaging Centre, Oxford, United Kingdom, Cardiovascular Clinical Research Facility, Oxford, United Kingdom, and Magnetic Resonance Imaging Unit, Department of Diagnostic Radiology, University of Hong Kong. Dr K.-K. Lau obtained funding, collected data, did the statistical analysis and interpretation, and wrote and revised the article. Drs L. Li, C.E. Lovelock, G. Zamboni, T.-T. Chan, M.-F. Chiang, and K.-T. Lo collected data. Dr Küker provided study supervision and acquired data. Dr Mak provided study supervision and funding, acquired and interpreted imaging data, and revised the manuscript. Dr Rothwell conceived and designed the overall study, provided study supervision and funding, acquired, analysed, and interpreted the data, and wrote and revised the manuscript.

## Sources of Funding

Oxford Vascular Study has been funded by the Wellcome Trust, Wolfson Foundation, UK Stroke Association, British Heart Foundation, Dunhill Medical Trust, National Institute for Health Research (NIHR), Medical Research Council, and the NIHR Oxford Biomedical Research Centre. Magnetic Resonance Imaging studies from University of Hong Kong (HKU) have been funded by the SK Yee Medical Foundation and HKU Strategic Research Theme in Neurosciences. Dr Rothwell is in receipt of an NIHR Senior Investigator Award and a Wellcome Trust Senior Investigator Award. Dr K.-K. Lau is funded by a University of Oxford Croucher Scholarship.

## Disclosures

None

## Supplementary Material
