# Supplementary material for: Clinical Correlates, Ethnic Differences, and Prognostic Implications of Perivascular Spaces in Transient Ischemic Attack and Ischemic Stroke
Source: Stroke. 2017 May 22;48(6):1470–7. doi: 10.1161/STROKEAHA.117.016694 (PMC5436733; doi:10.1161/STROKEAHA.117.016694)
Supplement: Supplementary file 1 [file str-48-1470-s001.pdf]

<u>Supplementary Materials</u>

Supplementary Tables:

Supplementary Table I. Imaging sequence parameters of the OXVASC and HKU cohorts

Supplementary Table II. Aetiology of TIA/ischaemic strokes according to TOAST classification

Supplementary Table III. Proportion of TIA/ischaemic stroke patients with perivascular spaces according to TOAST classification

Supplementary Table IV. Clinical and imaging characteristics in OXVASC and HKU patients who were scanned by a 3T MRI

Supplementary Table V. Clinical and imaging correlates of TIA/'ischaemic stroke patients with increasing burden of perivascular spaces

Supplementary Table VI. Clinical correlates of >20 perivascular spaces

Supplementary Table VII. Cox regression analyses of risk of death in patients with increasing burden of perivascular spaces vs. <11 perivascular spaces

Supplementary Table VIII. Cox regression analyses of recurrent stroke with increasing burden of perivascular spaces vs. <11 perivascular spaces

Supplementary Table IX. Cox regression analyses of risk of recurrent stroke with increasing burden of perivascular spaces vs. <11 perivascular spaces, stratified by OXVASC MRI scanner

Supplementary Table X. Cox regression analyses of risk of recurrent stroke with increasing burden of perivascular spaces vs. <11 perivascular spaces, stratified by white matter disease burden

Supplementary Table XI. Cox regression analysis of risk of recurrent ischaemic stroke with increasing burden of small vessel disease

The severity of white matter disease was determined for each patient according to the Fazekas scale.[1] Subcortical white matter hyperintensity (WMH) was graded as 0 (absent), 1 (puntate foci), 2 (beginning confluence of foci) and 3 (large confluent areas); whilst periventricular WMH was graded as 0 (no WMH except for small triangular foci surrounding the frontal horns), 1 (periventricular hyperintensity surrounding the anterior and posterior horns ± discrete WMHs), 2 (extensive patchy WMHs and their early confluent stages), and 3 (confluent, completely surrounding lateral ventricles). Cerebral microbleeds were defined as rounded, hypodense foci up to 10mm in size and were differentiated from microbleed mimics based on current guidelines.[2] The location and number of microbleeds were scored according to the Microbleed Anatomical Rating Scale and the location and number of microbleeds were recorded.[3] Lacunes were defined as rounded or ovoid lesions, >3 and <20mm in diameter, in the BG, internal capsule, CS or brainstem, of cerebrospinal fluid signal density on T2 and FLAIR and no increased signal on diffused weighted imaging.[4]

The definition of recurrent stroke required a sudden new neurological deficit fitting the definition of ischaemic stroke, or intracerebral haemorrhage, occurring after a period of unequivocal neurological stability and not attributable to cerebral oedema, mass effect, or haemorrhagic transformation of the incident cerebral infarction. Vascular death was defined as death due to lethal cardiac arrhythmias, acute coronary syndrome, congestive heart failure, fatal stroke, pulmonary embolism, aortic dissection or unexplained sudden death.

Supplementary References

1. Fazekas F, Chawluk JB, Alavi A, Hurtig HI, Zimmerman RA. Mr signal abnormalities at 1.5 t in alzheimer's dementia and normal aging. *AJR Am J Roentgenol*. 1987;149:351-356
2. Greenberg SM, Vernooij MW, Cordonnier C, Viswanathan A, Al-Shahi Salman R, Warach S, et al. Cerebral microbleeds: A guide to detection and interpretation. *Lancet Neurol*. 2009;8:165-174
3. Gregoire SM, Chaudhary UJ, Brown MM, Yousry TA, Kallis C, Jager HR, et al. The microbleed anatomical rating scale (mars): Reliability of a tool to map brain microbleeds. *Neurology*. 2009;73:1759-1766
4. Wardlaw JM, Smith EE, Biessels GJ, Cordonnier C, Fazekas F, Frayne R, et al. Neuroimaging standards for research into small vessel disease and its contribution to ageing and neurodegeneration. *Lancet Neurol*. 2013;12:822-838

Supplementary Table I. Imaging sequence parameters of the OXVASC and HKU cohorts

MR parameters	HKU<br>Achieva, Philips Healthcare	OXVASC scanner 1<br>Magnetom Verio, Siemens Healthcare	OXVASC scanner 2<br>Discovery MR750, GE Healthcare	OXVASC scanner 3<br>Achieva, Philips Healthcare	OXVASC scanner 4<br>Signa HDxt, GE Healthcare
**Patients scanned**	1003	388	62	493	137
**Field strength (T)**	3	3	3	1.5	1.5
**T1W TR/TE/TI (ms)**	2000/20/800	2000/1.94/880	-	701/16	-
**T2W TR/TE (ms)**	2377/80	6000/96	5800/94	5061/100	3760/100
**FLAIR TR/TE/TI (ms) (3D)**	4800/282/1650	9000/88/2500	9600/130/2350	11000/140/2800	8080/112/2200
**Diffusion TR/TE (ms)**	2874/46	5300/91	6000/84	2891/73	6100/71
**GRE / SWI TR/TE (ms) (3D)**	SWI 28/23	GRE 504/15	GRE 500/20	GRE 694/23	GRE 560/25
**Pixel bandwidth (Hz)**	218.5 (T1W)<br>350.7 (T2W)<br>144.7 (FLAIR)<br>40.2 (Diffusion)<br>455.7 (SWI)	240 (T1W)<br>220 (T2W)<br>202 (FLAIR)<br>1374 (Diffusion)<br>200 (GRE)	-<br>50 (T2W)<br>41.7 (FLAIR)<br>250 (Diffusion)<br>31.3 (GRE)	87.4 (T1W)<br>88.5 (T2W)<br>375 (FLAIR)<br>25.3 (Diffusion)<br>109.3 (GRE)	-<br>47.6 (T2W)<br>31.3 (FLAIR)<br>-<br>75 (GRE)
**Matrix**	308x207 (T1W)<br>308x235 (T2W)<br>228x227 (FLAIR)<br>112x87 (Diffusion)<br>256x224 (SWI)	256x256 (T1W)<br>320x320 (T2W)<br>192x192 (FLAIR)<br>130x130 (Diffusion)<br>320x256 (GRE)	-<br>512 (T2W)<br>384x224 (FLAIR)<br>128x128 (Diffusion)<br>288x224 (GRE)	118x214 (T1W)<br>356x193 (T2W)<br>236x159 (FLAIR)<br>97x84 (Diffusion)<br>256x163 (GRE)	416x256 (T2W)<br>256x224 (FLAIR)<br>128x128 (Diffusion)<br>288x192 (GRE)
**No. of slices**	25 (T1W)<br>25 (T2W)<br>30 (FLAIR)<br>25 (Diffusion)<br>25 (SWI)	208 (T1W)<br>25 (T2W)<br>50 (FLAIR)<br>25 (Diffusion)<br>25 (GRE)	25	25 (T1W)<br>25 (T2W)<br>28 (FLAIR)<br>25 (Diffusion)<br>22 (GRE)	25
**Slice thickness (mm)**	5 (T1W)<br>5 (T2W)<br>5 (FLAIR coronal)<br>2.5 (FLAIR axial)<br>5 (Diffusion)<br>5 (SWI)	1 (T1W)<br>5 (T2W)<br>3 (FLAIR)<br>5 (Diffusion)<br>5 (GRE)	5	5	5
**Inter-slice gap (mm)**	0.5 (T1W)<br>0.5(T2W)<br>0.5 (FLAIR coronal)<br>0 (FLAIR axial)<br>0.5 (Diffusion)<br>0.5 (SWI)	0 (T1W)<br>1 (T2W)<br>0 (FLAIR coronal)<br>1 (Diffusion)<br>1 (GRE)	1	1	1
**Voxel size (mm^3)**	0.75x0.95x5.0 (T1W)<br>0.75x0.76x5.0 (T2W)<br>1.10x1.10x1.12 (FLAIR)<br>2.05x2.64x5.0 (Diffusion)<br>0.90x0.90x2.00 (SWI)	1.0x1.0x1.0 (T1W)<br>0.8x0.8x5.0 (T2W)<br>1.0x1.0x3.0 (FLAIR)<br>1.8x1.8x5.0 (Diffusion)<br>0.9x0.8x5.0 (GRE)	-	0.53x0.53x5.0 (T1W)<br>0.65x0.65x5.0 (T2W)<br>0.82x0.81x5.0 (FLAIR)<br>1.74x1.73x5.0 (Diffusion)<br>0.90x0.90x5.0 (GRE)	-

Supplementary Table II. Aetiology of TIA/ischaemic strokes according to TOAST classification

	OXVASC, UK n=1028 (542 TIA, 486 ischaemic stroke)	HKU, HK n=974 (974 ischaemic stroke)	All n=2002
Small vessel disease (%)	124 (12.1)	413 (42.4)	537 (26.8)
Large artery atherosclerosis (%)	137 (13.3)	334 (34.3)	471 (23.5)
Cardio-embolic (%)	160 (15.6)	118 (12.1)	278 (13.9)
Undetermined (%)	514 (50.0)	42 (4.3)	556 (27.8)
Multiple (%)	35 (3.4)	28 (2.9)	63 (2.1)
Unknown (%)	26 (2.5)	22 (2.3)	48 (2.4)
Others (%)	32 (3.1)	17 (1.7)	49 (2.4)

Supplementary Table III. Proportion of TIA/ischaemic stroke patients with perivascular spaces according to TOAST classification

	OXVASC n=1028	HKU n=974	p
**Small vessel disease**	n=124	n=413	
N with basal ganglia PVSs (%)			
<11 (%)	53 (42.7)	269 (65.1)	p<0.0001
11-20 (%)	39 (31.5)	109 (26.4)	
>20 (%)	32 (25.8)	35 (8.5)	
N with centrum semi-ovale PVSs (%)			
<11 (%)	15 (12.1)	172 (41.6)	p<0.0001
11-20 (%)	39 (31.5)	200 (48.4)	
>20 (%)	70 (56.5)	41 (9.9)	
**Large artery atherosclerosis**	n=137	n=334	
N with basal ganglia PVSs (%)			
<11 (%)	52 (38.0)	232 (69.5)	p<0.0001
11-20 (%)	46 (33.6)	82 (24.6)	
>20 (%)	39 (28.5)	20 (6.0)	
N with centrum semi-ovale PVSs (%)			
<11 (%)	17 (12.4)	134 (40.1)	p<0.0001
11-20 (%)	40 (29.2)	162 (48.5)	
>20 (%)	80 (58.4)	38 (11.4)	
**Cardio-embolic**	n=160	n=118	
N with basal ganglia PVSs (%)			
<11 (%)	66 (41.3)	76 (64.4)	p<0.0001
11-20 (%)	42 (26.3)	33 (28.0)	
>20 (%)	52 (32.5)	9 (7.6)	
N with centrum semi-ovale PVSs (%)			
<11 (%)	27 (16.9)	56 (47.5)	p<0.0001
11-20 (%)	48 (30.0)	47 (39.8)	
>20 (%)	85 (53.1)	15 (12.7)	

PVS=perivascular space

Supplementary Table IV. Clinical and imaging characteristics in OXVASC and HKU patients who were scanned by a 3T MRI

	OXVASC, UK<br>n=446<br>(267 TIA , 179 ischaemic stroke)	HKU, HK<br>n=974<br>974 ischaemic stroke	p
**Baseline clinical characteristics**			
Mean age, yr (SD)	70 (14)	69 (12)	0.39
Males (%)	235 (52.7)	583 (59.9)	0.011
Hypertension (%)	237 (53.1)	640 (65.7)	<0.0001
Diabetes (%)	53 (11.9)	275 (28.2)	<0.0001
Hyperlipidaemia (%)	154 (34.5)	249 (25.6)	0.001
Ever-smokers (%)	190 (42.6)	291 (29.9)	<0.0001
Atrial fibrillation (%)	69 (15.5)	128 (13.1)	0.24
Prior TIA/stroke (%)	77 (17.3)	154 (15.8)	0.49
**Imaging characteristics**			
N with DWI positive lesion (%)	101 (22.6)	759 (77.9)	<0.0001
N with basal ganglia PVSs (%)			
<11 (%)	181 (40.6)	659 (67.7)	<0.0001
11-20 (%)	150 (33.6)	246 (25.3)	
>20 (%)	115 (25.8)	69 (7.1)	
N with centrum semi-ovale PVSs (%)			
<11 (%)	60 (13.5)	410 (42.1)	<0.0001
11-20 (%)	138 (30.9)	463 (47.5)	
>20 (%)	248 (55.6)	101 (10.4)	
N with lacunes (%)	58 (13.0)	430 (44.1)	<0.0001
N with microbleeds (%)	64 (14.3)	441 (45.3)	<0.0001
1 microbleed (%)	35 (7.8)	179 (18.4)	
2-4 microbleeds (%)	17 (3.8)	145 (14.9)	
≥5 microbleeds (%)	12 (2.7)	117 (12.0)	
N with periventricular WMH (%)			
Grade 1 (%)	182 (40.8)	213 (21.9)	<0.0001
Grade 2 (%)	89 (20.0)	75 (7.7)	
Grade 3 (%)	31 (7.0)	30 (3.1)	
N with subcortical WMH (%)			
Grade 1 (%)	141 (31.6)	475 (48.8)	<0.0001
Grade 2 (%)	70 (15.7)	278 (28.5)	
Grade 3 (%)	53 (11.9)	155 (15.9)	

TIA=transient ischaemic attack; DWI=diffusion weighted imaging; PVS=perivascular space; WMH=white matter hyperintensity

Supplementary Table V. Clinical and imaging correlates of TIA/ischaemic stroke patients with increasing burden of perivascular spaces

	Number of basal ganglia PVSs				Number of centrum semi-ovale PVSs			
	**<11 n=1186**	**11-20 n=517**	**>20 n=299**	p_{trend}	**<11 n=636**	**11-20 n=794**	**>20 n=572**	p_{trend}
Age, yr (SD)	64 (13)	74 (10)	77 (9)	<0.0001	65 (14)	69 (13)	73 (11)	<0.0001
Male sex (%)	679 (57.3)	291 (56.3)	151 (50.5)	0.11	360 (56.6)	472 (59.4)	289 (50.5)	0.004
Hypertension (%)	645 (54.4)	360 (69.6)	198 (66.2)	<0.0001	337 (53.0)	504 (63.5)	362 (63.3)	<0.0001
Diabetes (%)	247 (20.8)	110 (21.3)	54 (18.1)	0.51	136 (21.4)	174 (21.9)	101 (17.7)	0.13
Hyperlipidaemia (%)	366 (30.9)	166 (32.1)	98 (32.8)	0.76	186 (29.2)	241 (30.4)	203 (35.5)	0.045
Ever-smoker (%)	485 (40.9)	208 (40.2)	119 (39.8)	0.92	249 (39.2)	314 (39.6)	249 (43.5)	0.23
Atrial fibrillation (%)	123 (10.4)	93 (18.0)	72 (24.1)	<0.0001	88 (13.8)	100 (12.6)	100 (17.5)	0.035
GFR<60ml/min/1.73m^2	210 (17.9)	145 (28.4)	89 (29.9)	<0.0001	124 (19.7)	173 (22.0)	147 (25.9)	0.033
Severe periventricular WMH (%)	30 (2.5)	30 (5.8)	66 (22.1)	<0.0001	27 (4.2)	33 (4.2)	66 (11.5)	<0.0001
Severe subcortical WMH (%)	87 (7.3)	91 (17.6)	94 (31.4)	<0.0001	68 (10.7)	99 (12.5)	105 (18.4)	0.0003
≥5 Microbleeds (%)	58 (4.9)	47 (9.1)	51 (17.1)	<0.0001	63 (9.9)	57 (7.2)	36 (6.3)	0.046
Lacunes (%)	337 (28.4)	176 (34.0)	99 (33.1)	0.040	184 (28.9)	269 (33.9)	159 (27.8)	0.031

PVS=perivascular space, GFR=glomerular filtration rate, WMH=white matter hyperintensity

Supplementary Table VI. Clinical correlates of >20 perivascular spaces

	Univariate						Age and sex adjusted		Multi-variate[†] adjusted	
**Basal ganglia**	**OXVASC OR (95% CI)**	**p**	**HKU OR (95% CI)**	**p**	**Combined[*] OR (95% CI)**	**p**	**Combined[*] OR (95% CI)**	**p**	**Combined[*] OR (95% CI)**	**p**
Age	1.08 (1.06-1.10)	<0.0001	1.09 (1.06-1.12)	<0.0001	1.08 (1.07-1.10)	<0.0001	1.08 (1.07-1.10)	<0.0001	1.05 (1.03-1.07)	<0.0001
Male sex	0.79 (0.59-1.06)	0.12	0.98 (0.60-1.62)	0.94	0.84 (0.65-1.08)	0.17	1.03 (0.78-1.34)	0.86	1.17 (0.86-1.59)	0.33
Hypertension	1.71 (1.26-2.31)	0.001	1.30 (0.76-2.23)	0.34	1.60 (1.23-2.09)	0.001	1.09 (0.82-1.44)	0.57	0.98 (0.70-1.37)	0.91
Diabetes	1.41 (0.94-2.12)	0.096	0.75 (0.42-1.34)	0.34	1.13 (0.81-1.57)	0.48	1.00 (0.71-1.41)	1.00	1.09 (0.74-1.62)	0.67
Hyperlipidaemia	1.07 (0.79-1.45)	0.67	0.47 (0.24-0.94)	0.032	0.91 (0.70-1.20)	0.51	0.78 (0.59-1.04)	0.095	0.81 (0.58-1.13)	0.22
Ever-smoker	0.77 (0.57-1.03)	0.08	0.58 (0.32-1.06)	0.07	0.73 (0.56-0.94)	0.017	0.82 (0.62-1.10)	0.19	0.86 (0.62-1.19)	0.37
Atrial fibrillation	2.46 (1.72-3.54)	<0.0001	1.43 (0.75-2.75)	0.28	2.15 (1.57-2.93)	<0.0001	1.35 (0.97-1.87)	0.078	1.58 (1.10-2.29)	0.014
GFR<60ml/min/1.73m^2	1.31 (0.93-1.82)	0.12	2.50 (1.49-4.18)	0.001	1.57 (1.18-2.08)	0.002	0.76 (0.55-1.04)	0.081	0.81 (0.57-1.15)	0.24
Periventricular WMH	3.14 (2.62-3.76)	<0.0001	2.72 (2.12-3.48)	<0.0001	2.99 (2.58-3.45)	<0.0001	2.43 (2.08-2.84)	<0.0001	2.01 (1.66-2.44)	<0.0001
Subcortical WMH	2.61 (2.23-3.07)	<0.0001	2.55 (1.89-3.45)	<0.0001	2.60 (2.26-3.00)	<0.0001	2.16 (1.86-2.50)	<0.0001	1.44 (1.20-1.72)	<0.0001
Deep microbleed number	1.23 (1.01-1.50)	0.037	1.05 (1.02-1.09)	0.002	1.06 (1.03-1.10)	0.001	1.07 (1.03-1.11)	<0.0001	1.05 (1.00-1.10)	0.063
Lobar microbleed number	1.05 (1.00-1.10)	0.042	1.02 (1.00-1.03)	0.014	1.02 (1.01-1.04)	0.002	1.03 (1.01-1.04)	0.0004	1.00 (0.98-1.02)	0.84
Lacunes	3.22 (2.29-4.54)	<0.0001	1.64 (0.97-2.74)	0.063	1.87 (1.40-2.49)	<0.0001	1.67 (1.24-2.26)	0.001	1.18 (0.84-1.65)	0.34
**Centrum semiovale**										
Age	1.05 (1.04-1.06)	<0.0001	1.01 (1.00-1.03)	0.17	1.04 (1.03-1.05)	<0.0001	1.04 (1.03-1.05)	<0.0001	1.01 (1.00-1.03)	0.020
Male sex	0.78 (0.61-1.00)	0.052	0.89 (0.59-1.35)	0.59	0.81 (0.66-1.00)	0.052	0.89 (0.72-1.11)	0.32	0.88 (0.69-1.12)	0.29
Hypertension	1.64 (1.28-2.10)	<0.0001	1.41 (0.89-2.23)	0.14	1.59 (1.27-1.97)	<0.0001	1.23 (0.97-1.54)	0.085	1.13 (0.87-1.48)	0.36
Diabetes	1.17 (0.82-1.68)	0.39	1.32 (0.85-2.05)	0.22	1.23 (0.93-1.63)	0.14	1.12 (0.84-1.49)	0.43	1.15 (0.83-1.60)	0.39
Hyperlipidaemia	1.01 (0.78-1.30)	0.96	1.12 (0.71-1.78)	0.63	1.04 (0.83-1.29)	0.77	0.92 (0.73-1.16)	0.47	0.94 (0.72-1.23)	0.67
Ever-smoker	0.77 (0.60-0.98)	0.034	0.84 (0.53-1.34)	0.48	0.78 (0.63-0.97)	0.027	0.88 (0.70-1.11)	0.29	0.91 (0.70-1.17)	0.45
Atrial fibrillation	1.60 (1.14-2.24)	0.007	0.79 (0.41-1.53)	0.49	1.35 (1.01-1.80)	0.043	0.98 (0.72-1.32)	0.87	0.85 (0.61-1.19)	0.34
GFR<60ml/min/1.73m^2	1.26 (0.94-1.68)	0.12	1.40 (0.87-2.25)	0.17	1.30 (1.01-1.66)	0.041	0.81 (0.61-1.06)	0.12	0.87 (0.64-1.18)	0.37
Periventricular WMH	1.90 (1.65-2.19)	<0.0001	1.00 (0.77-1.31)	1.00	1.62 (1.45-1.82)	<0.0001	1.35 (1.18-1.53)	<0.0001	0.90 (0.76-1.08)	0.26
Subcortical WMH	1.87 (1.64-2.14)	<0.0001	1.28 (1.00-1.63)	0.047	1.71 (1.53-1.92)	<0.0001	1.48 (1.31-1.67)	<0.0001	1.28 (1.09-1.50)	0.003
Deep microbleed number	1.25 (0.99-1.58)	0.056	0.98 (0.91-1.05)	0.52	1.00 (0.96-1.04)	0.97	1.00 (0.96-1.04)	0.98	0.95 (0.86-1.04)	0.25
Lobar microbleed number	1.02 (0.98-1.05)	0.38	0.98 (0.92-1.03)	0.39	1.00 (0.98-1.02)	0.87	1.00 (0.98-1.02)	0.74	0.98 (0.94-1.02)	0.26
Lacunes	1.89 (1.37-2.62)	0.0001	1.39 (0.92-2.10)	0.12	1.68 (1.30-2.17)	<0.0001	1.55 (1.19-2.01)	0.001	1.26 (0.95-1.69)	0.11

[*]Adjusted for Centre
[†]Also adjusted for MRI scanner strength, CS-PVSs for prediction of BG-PVSs and BG-PVSs for prediction of CS-PVSs
OR=odds ratio, CI=confidence interval, GFR=glomerular filtration rate, WMH=white matter hyperintensity, PVS=perivascular space

Supplementary Table VII. Cox regression analyses of risk of death in patients with increasing burden of perivascular spaces vs. <11 perivascular spaces

	Unadjusted HR (95% CI)*			HR (95% CI) adjusted for age and sex *			HR (95% CI) adjusted for age, sex, vascular risk factors† and MRI scanner strength*		
**Number of PVSs:**	**11-20**	**>20**	p_{trend}	**11-20**	**>20**	p_{trend}	**11-20**	**>20**	p_{trend}
**Basal ganglia PVSs**									
**All-cause mortality**									
OXVASC	1.68 (1.10-2.58)	3.23 (2.20-4.74)	<0.0001	0.81 (0.52-1.26)	1.42 (0.94-2.13)	0.089	0.74 (0.47-1.17)	1.25 (0.83-1.89)	0.27
HKU	1.59 (1.08-2.35)	2.67 (1.55-4.61)	0.0001	1.03 (0.69-1.54)	1.36 (0.78-2.39)	0.37	1.04 (0.69-1.55)	1.32 (0.75-2.31)	0.41
Combined‡	1.63 (1.23-2.18)	3.04 (2.24-4.13)	<0.0001	0.92 (0.68-1.24)	1.44 (1.05-1.99)	0.058	0.89 (0.66-1.20)	1.33 (0.96-1.85)	0.16
**Vascular death**									
OXVASC	1.53 (0.63-3.71)	3.32 (1.55-7.12)	0.002	0.79 (0.32-2.00)	1.55 (0.68-3.50)	0.28	0.73 (0.29-1.85)	1.31 (0.58-3.00)	0.49
HKU	1.66 (0.94-2.94)	2.54 (1.12-5.77)	0.011	1.03 (0.58-1.85)	1.22 (0.53-2.83)	0.68	1.06 (0.59-1.93)	1.21 (0.52-2.81)	0.67
Combined‡	1.62 (1.01-2.62)	2.98 (1.74-5.12)	<0.0001	0.95 (0.58-1.55)	1.43 (0.81-2.50)	0.31	0.93 (0.57-1.54)	1.31 (0.74-2.31)	0.47
**Nonvascular death**									
OXVASC	1.62 (0.96-2.73)	2.95 (1.84-4.75)	<0.0001	0.80 (0.46-1.37)	1.33 (0.80-2.20)	0.28	0.72 (0.41-1.24)	1.17 (0.70-1.95)	0.55
HKU	1.53 (0.90-2.62)	2.79 (1.34-5.78)	0.005	1.03 (0.60-1.77)	1.50 (0.71-3.18)	0.40	1.01 (0.58-1.76)	1.42 (0.67-3.02)	0.47
Combined‡	1.58 (1.09-2.29)	2.80 (1.91-4.10)	<0.0001	0.90 (0.61-1.32)	1.39 (0.93-2.07)	0.17	0.86 (0.58-1.27)	1.32 (0.87-2.00)	0.30
**Centrum semi-ovale PVSs**									
**All-cause mortality**									
OXVASC	1.08 (0.67-1.74)	1.90 (1.23-2.92)	0.001	0.54 (0.33-0.89)	0.78 (0.50-1.22)	0.75	0.50 (0.30-0.81)	0.68 (0.43-1.07)	0.40
HKU	0.80 (0.56-1.16)	0.53 (0.26-1.06)	0.053	0.81 (0.56-1.17)	0.49 (0.24-0.98)	0.035	0.87 (0.60-1.27)	0.50 (0.25-1.01)	0.068
Combined‡	0.90 (0.67-1.20)	1.28 (0.94-1.76)	0.17	0.69 (0.51-0.93)	0.77 (0.55-1.07)	0.11	0.70 (0.52-0.94)	0.74 (0.53-1.03)	0.072
**Vascular death**									
OXVASC	1.06 (0.37-3.07)	2.57 (1.03-6.45)	0.020	0.54 (0.18-1.63)	1.11 (0.43-2.88)	0.41	0.49 (0.16-1.48)	0.95 (0.36-2.48)	0.59
HKU	0.89 (0.52-1.51)	0.40 (0.12-1.33)	0.17	0.90 (0.53-1.53)	0.36 (0.11-1.20)	0.14	0.99 (0.57-1.71)	0.36 (0.11-1.20)	0.19
Combined‡	0.91 (0.57-1.47)	1.27 (0.73-2.20)	0.50	0.77 (0.48-1.25)	0.85 (0.47-1.52)	0.50	0.80 (0.49-1.30)	0.81 (0.45-1.45)	0.43
**Nonvascular death**									
OXVASC	0.91 (0.52-1.60)	1.52 (0.91-2.53)	0.073	0.47 (0.26-0.83)	0.63 (0.37-1.08)	0.29	0.43 (0.24-0.77)	0.56 (0.33-0.95)	0.14
HKU	0.74 (0.45-1.22)	0.63 (0.26-1.49)	0.17	0.74 (0.45-1.22)	0.58 (0.24-1.40)	0.14	0.78 (0.47-1.31)	0.61 (0.26-1.47)	0.20
Combined‡	0.81 (0.56-1.18)	1.20 (0.80-1.78)	0.47	0.61 (0.41-0.89)	0.70 (0.46-1.07)	0.091	0.61 (0.42-0.90)	0.68 (0.44-1.03)	0.067

*Compared with <11 PVSs as reference
†Hypertension, hyperlipidaemia, diabetes mellitus, atrial fibrillation, smoking history
‡Also adjusted for Centre
HR=hazard ratio, CI=confidence interval, ICH=intracerebral haemorrhage

Supplementary Table VIII. Cox regression analyses of recurrent stroke with increasing burden of perivascular spaces vs. <11 perivascular spaces

	Unadjusted HR (95% CI)*			HR (95% CI) adjusted for age and sex*			HR (95% CI) adjusted for age, sex, vascular risk factors† and MRI scanner strength*		
**Number of PVSs:**	**11-20**	**>20**	**p_{trend}**	**11-20**	**>20**	**p_{trend}**	**11-20**	**>20**	**p_{trend}**
**Basal ganglia PVSs**									
**Recurrent stroke**									
OXVASC	1.36 (0.79-2.32)	2.45 (1.52-3.95)	0.0003	1.16 (0.65-2.05)	2.03 (1.19-3.46)	0.01	1.11 (0.62-1.97)	1.90 (1.11-3.25)	0.021
HKU	1.46 (0.95-2.24)	2.95 (1.70-5.11)	0.0002	1.11 (0.72-1.73)	1.97 (1.11-3.49)	0.05	1.20 (0.77-1.87)	2.32 (1.29-4.15)	0.013
Combined‡	1.40 (1.00-1.95)	2.62 (1.83-3.77)	<0.0001	1.11 (0.78-1.57)	1.92 (1.30-2.83)	0.003	1.13 (0.79-1.61)	1.94 (1.31-2.89)	0.002
**Ischaemic stroke**									
OXVASC	1.24 (0.70-2.20)	2.36 (1.43-3.89)	0.001	1.11 (0.60-2.04)	2.08 (1.18-3.65)	0.012	1.04 (0.56-1.92)	1.99 (1.13-3.49)	0.020
HKU	1.56 (0.98-2.47)	2.38 (1.24-4.58)	0.004	1.15 (0.72-1.86)	1.55 (0.79-3.04)	0.22	1.30 (0.80-2.11)	1.94 (0.98-3.85)	0.056
Combined‡	1.40 (0.98-2.01)	2.39 (1.61-3.54)	<0.0001	1.12 (0.77-1.63)	1.77 (1.16-2.70)	0.014	1.15 (0.78-1.68)	1.82 (1.18-2.80)	0.011
**Intracerebral haemorrhage**									
OXVASC	2.55 (0.51-12.69)	3.31 (0.66-16.59)	0.13	1.39 (0.25-7.75)	1.63 (0.29-9.35)	0.58	1.03 (0.17-6.16)	0.95 (0.14-6.22)	0.95
HKU	1.01 (0.32-3.17)	5.00 (1.73-14.43)	0.019	0.87 (0.27-2.83)	4.07 (1.30-12.76)	0.062	0.79 (0.24-2.58)	3.84 (1.20-12.29)	0.089
Combined‡	1.33 (0.54-3.29)	4.02 (1.63-9.94)	0.007	1.02 (0.40-2.63)	2.77 (1.05-7.35)	0.074	0.95 (0.37-2.46)	2.58 (0.97-6.89)	0.10
**Centrum semiovale PVSs**									
**Recurrent stroke**									
OXVASC	1.07 (0.58-1.98)	1.84 (1.06-3.19)	0.016	0.90 (0.48-1.70)	1.43 (0.79-2.59)	0.13	0.83 (0.44-1.57)	1.30 (0.71-2.37)	0.21
HKU	1.02 (0.69-1.50)	0.39 (0.15-0.97)	0.15	1.02 (0.70-1.50)	0.37 (0.15-0.92)	0.12	1.07 (0.73-1.58)	0.37 (0.15-0.94)	0.16
Combined‡	1.02 (0.73-1.41)	1.15 (0.78-1.68)	0.52	0.92 (0.66-1.28)	0.91 (0.61-1.35)	0.60	0.92 (0.66-1.28)	0.89 (0.60-1.33)	0.57
**Ischaemic stroke**									
OXVASC	1.20 (0.63-2.26)	1.72 (0.95-3.10)	0.053	1.04 (0.54-2.01)	1.41 (0.75-2.67)	0.21	0.94 (0.49-1.82)	1.28 (0.68-2.41)	0.33
HKU	1.04 (0.68-1.59)	0.29 (0.09-0.93)	0.14	1.04 (0.68-1.59)	0.27 (0.08-0.87)	0.11	1.12 (0.73-1.72)	0.28 (0.09-0.92)	0.18
Combined‡	1.07 (0.75-1.52)	1.07 (0.70-1.63)	0.73	0.96 (0.67-1.37)	0.85 (0.55-1.31)	0.46	0.96 (0.67-1.37)	0.83 (0.54-1.28)	0.42
**Intracerebral haemorrhage**									
OXVASC	-	2.89 (0.59-14.17)	0.087	-	1.37 (0.25-7.46)	0.28	-	1.29 (0.19-8.69)	0.35
HKU	0.90 (0.36-2.27)	0.83 (0.18-3.83)	0.77	0.90 (0.36-2.26)	0.80 (0.17-3.71)	0.75	0.86 (0.34-2.17)	0.75 (0.16-3.51)	0.67
Combined‡	0.71 (0.30-1.72)	1.65 (0.65-4.20)	0.43	0.67 (0.28-1.61)	1.35 (0.51-3.57)	0.70	0.69 (0.28-1.67)	1.36 (0.51-3.59)	0.69

*Compared with <11 PVSs as reference
†Hypertension, hyperlipidaemia, diabetes mellitus, atrial fibrillation, smoking history
‡Also adjusted for Centre
HR=hazard ratio, CI=confidence interval, PVS=perivascular space

Supplementary Table IX. Cox regression analyses of risk of recurrent stroke with increasing burden of perivascular spaces vs. <11 perivascular spaces, stratified by OXVASC MRI scanner

	**Unadjusted HR (95% CI)***			**HR (95% CI) adjusted for age and sex***		
**Number of PVSs:**	**11-20**	**>20**	**p_{trend}**	**11-20**	**>20**	**p_{trend}**
**Basal ganglia PVSs**						
OXVASC scanner 1 (3T)	1.20 (0.35-4.15)	3.06 (1.05-8.96)	0.035	1.07 (0.29-4.01)	2.60 (0.77-8.83)	0.090
OXVASC scanner 2 (3T)	2.69 (0.38-19.13)	10.82 (2.06-56.90)	0.004	2.67 (0.34-20.71)	17.60 (2.11-146.52)	0.009
OXVASC scanner 3 (1.5T)	4.43 (1.13-17.28)	1.11 (0.13-9.54)	0.37	2.67 (0.59-12.05)	0.62 (0.07-5.85)	0.94
OXVASC scanner 4 (1.5T)	1.11 (0.54-2.31)	1.94 (1.02-3.67)	0.056	0.97 (0.44-2.12)	1.66 (0.81-3.38)	0.19
**Centrum semi-ovale PVSs**						
OXVASC scanner 1 (3T)	0.66 (0.06-7.33)	3.19 (0.42-24.08)	0.037	0.56 (0.05-6.28)	2.54 (0.32-20.03)	0.057
OXVASC scanner 2 (3T)	0.76 (0.13-4.56)	2.67 (0.58-12.23)	0.22	0.66 (0.11-4.09)	2.26 (0.48-10.70)	0.30
OXVASC scanner 3 (1.5T)	3.39 (0.66-17.52)	4.05 (0.64-25.64)	0.11	2.39 (0.42-13.65)	1.97 (0.26-14.82)	0.53
OXVASC scanner 4 (1.5T)	1.04 (0.49-2.23)	1.50 (0.75-3.01)	0.21	0.90 (0.40-2.01)	1.21 (0.55-2.65)	0.54

*Compared with <11 PVSs as reference
HR=hazard ratio, CI=confidence interval, PVS=perivascular space

Supplementary Table X. Cox regression analyses of risk of recurrent stroke with increasing burden of perivascular spaces vs. <11 perivascular spaces, stratified by white matter disease burden

	Unadjusted HR (95% CI)*			HR (95% CI) adjusted for age and sex*			HR (95% CI) adjusted for age, sex, vascular risk factors† and MRI scanner strength*		
**Number of PVSs:**	**11-20**	**>20**	p_{trend}	**11-20**	**>20**	p_{trend}	**11-20**	**>20**	p_{trend}
**No or mild WMH**									
**Basal ganglia PVSs**									
OXVASC	0.67 (0.26-1.75)	2.47 (1.12-5.46)	0.13	0.55 (0.20-1.48)	1.92 (0.81-4.56)	0.38	0.65 (0.24-1.76)	2.17 (0.89-5.34)	0.24
HKU	1.27 (0.61-2.65)	3.84 (1.18-12.51)	0.074	0.92 (0.43-1.94)	2.63 (0.79-8.72)	0.44	0.99 (0.46-2.11)	2.88 (0.85-9.78)	0.33
Combined‡	0.97 (0.54-1.73)	2.89 (1.48-5.62)	0.023	0.73 (0.40-1.33)	1.99 (0.99-4.00)	0.34	0.80 (0.44-1.47)	2.20 (1.07-4.52)	0.20
**Centrum semi-ovale PVSs**									
OXVASC	1.12 (0.48-2.58)	1.99 (0.91-4.35)	0.071	0.97 (0.41-2.32)	1.64 (0.71-3.80)	0.20	0.90 (0.38-2.17)	1.87 (0.81-4.33)	0.11
HKU	1.01 (0.55-1.84)	0.92 (0.31-2.67)	0.92	0.97 (0.53-1.78)	0.90 (0.31-2.63)	0.85	0.93 (0.51-1.72)	0.83 (0.28-2.45)	0.72
Combined‡	1.04 (0.64-1.71)	1.49 (0.84-2.64)	0.21	0.92 (0.56-1.51)	1.19 (0.66-2.15)	0.65	0.88 (0.53-1.44)	1.27 (0.71-2.29)	0.54
**Moderate to severe WMH**									
**Basal ganglia PVSs**									
OXVASC	1.30 (0.60-2.81)	1.49 (0.72-3.09)	0.29	1.37 (0.62-3.06)	1.57 (0.74-3.36)	0.25	1.23 (0.55-2.76)	1.37 (0.64-2.95)	0.42
HKU	1.32 (0.76-2.30)	2.32 (1.20-4.51)	0.019	1.10 (0.62-1.96)	1.74 (0.87-3.50)	0.16	1.09 (0.61-1.95)	1.87 (0.91-3.84)	0.13
Combined‡	1.28 (0.82-2.01)	1.79 (1.09-2.94)	0.022	1.17 (0.73-1.86)	1.59 (0.95-2.66)	0.081	1.15 (0.72-1.83)	1.53 (0.91-2.59)	0.12
**Centrum semi-ovale PVSs**									
OXVASC	0.64 (0.25-1.62)	0.81 (0.35-1.84)	0.92	0.64 (0.25-1.64)	0.81 (0.35-1.88)	0.93	0.63 (0.25-1.62)	0.74 (0.32-1.73)	0.72
HKU	0.95 (0.57-1.56)	0.10 (0.01-0.76)	0.034	0.98 (0.60-1.62)	0.10 (0.01-0.73)	0.035	1.10 (0.67-1.85)	0.10 (0.01-0.75)	0.066
Combined‡	0.82 (0.52-1.28)	0.60 (0.35-1.04)	0.069	0.81 (0.52-1.26)	0.57 (0.33-1.00)	0.048	0.85 (0.54-1.33)	0.57 (0.33-1.00)	0.051

*Compared with <11 PVSs as reference

†Hypertension, hyperlipidaemia, diabetes mellitus, atrial fibrillation, smoking history

‡Also adjusted for Centre

HR=hazard ratio, CI=confidence interval, PVS=perivascular space, WMH=white matter hyperintensity

Supplementary Table XI. Cox regression analyses of risk of recurrent ischaemic stroke with increasing burden of neuroimaging markers of small vessel disease

	**Unadjusted HR (95% CI)**	**p_{trend}**	**Multi-variate adjusted HR (95% CI) (Forward stepwise)**	**p_{trend}**
**Number of BG-PVSs***				
11-20	1.40 (0.98-2.01)	<0.0001	1.22 (0.84-1.77)	0.001
>20	2.39 (1.61-3.54)		1.75 (1.17-2.63)	
**Number of CS-PVSs***				
11-20	1.07 (0.75-1.52)	0.73	-	
>20	1.07 (0.70-1.63)		-	
**Lacunes**†	1.42 (1.02-1.96)	0.036	-	
**Microbleed**†				
1 microbleed	1.72 (1.13-2.62)	0.001	1.67 (1.10-2.53)	0.001
2-4 microbleed	1.64 (1.00-2.69)		1.43 (0.87-2.35)	
≥5 microbleeds	2.14 (1.32-3.49)		1.76 (1.06-2.92)	
**Periventricular WMH**†				
Grade 1	1.48 (1.02-2.13)	<0.0001	-	
Grade 2	1.91 (1.22-2.97)		-	
Grade 3	2.57 (1.49-4.44)		-	
**Subcortical WMH**†				
Grade 1	1.14 (0.72-1.83)	0.001	1.04 (0.66-1.64)	0.17
Grade 2	2.36 (1.48-3.76)		1.85 (1.15-2.98)	
Grade 3	1.64 (0.95-2.85)		1.09 (0.60-1.98)	

*Compared with <11 PVSs as reference
†Compared with no lacunes, microbleeds or WMH as reference
HR=hazard ratio, CI=confidence interval, BG=basal ganglia, CS=centrum semi-ovale, PVS=perivascular space, WMH=white matter hyperintensity
